# Pathway-specific polygenic risk scores correlate with clinical status and Alzheimer’s-related biomarkers

**DOI:** 10.21203/rs.3.rs-2583037/v1

**Published:** 2023-03-01

**Authors:** Nicholas J. Schork, Jeremy A. Elman

**Affiliations:** Translational Genomics Research Institute; University of California, San Diego

**Keywords:** Alzheimer’s disease, dementia, genetic risk score, amyloid, tau, hippocampal volume

## Abstract

**Background:**

*APOE* is the largest genetic risk factor for sporadic Alzheimer’s disease (AD), but there is a substantial polygenic component as well. Polygenic risk scores (PRS) can summarize small effects across the genome but may obscure differential risk associated with different molecular processes and pathways. Variability at the genetic level may contribute to the extensive phenotypic heterogeneity of Alzheimer’s disease (AD). Here, we examine polygenic risk impacting specific pathways associated with AD and examined its relationship with clinical status and AD biomarkers of amyloid, tau, and neurodegeneration (A/T/N).

**Methods:**

A total of 1,411 participants from the Alzheimer’s Disease Neuroimaging Initiative (ADNI) with genotyping data were included. Sets of variants identified from a pathway analysis of AD GWAS summary statistics were combined into clusters based on their assigned pathway. We constructed pathway-specific PRSs for each participant and tested their associations with diagnostic status (AD vs cognitively normal), abnormal levels of amyloid and ptau (positive vs negative), and hippocampal volume. The *APOE* region was excluded from all PRSs, and analyses controlled for *APOE*-ε4 carrier status.

**Results:**

Thirteen pathway clusters were identified relating to categories such as immune response, amyloid precursor processing, protein localization, lipid transport and binding, tyrosine kinase, and endocytosis. Eight pathway-specific PRSs were significantly associated with AD dementia diagnosis. Amyloid-positivity was associated with endocytosis and fibril formation, response misfolded protein, and regulation protein tyrosine PRSs. Ptau positivity and hippocampal volume were both related to protein localization and mitophagy PRS, and ptau positivity was additionally associated with an immune signaling PRS. A global AD PRS showed stronger associations with diagnosis and all biomarkers compared to pathway PRSs, suggesting a strong synergistic effect of all loci contributing to the global AD PRS.

**Conclusions:**

Pathway PRS may contribute to understanding separable disease processes, but do not appear to add significant power for predictive purposes. These findings demonstrate that, although genetic risk for AD is widely distributed, AD-phenotypes may be preferentially associated with risk in specific pathways. Defining genetic risk along multiple dimensions at the individual level may help clarify the etiological heterogeneity in AD.

## Background

Alzheimer’s disease is known to be influenced by many genetically-mediated factors, with a heritability estimated to be 60–80% (1). Although autosomal dominant forms of AD are due to mutations in the APP, PSEN1, and PSEN2 genes, sporadic or late onset Alzheimer’s disease has a more complex genetic basis. The *APOE* gene represents the single largest genetic risk factor (2), but over the past decade or so a number of additional AD risk genes have been discovered (3–6), with the most recent AD GWAS identifying 75 risk loci (7). Finding effective treatment for AD remains elusive, and there is increasing focus on its heterogenous presentation, both clinically and at the level of pathobiological mechanisms. Ultimately, identifying the sources of genetic risk for AD may not only shed light on the pathobiology of the disease but also lead to novel drug targets.

While large-scale genome-wide association studies (GWAS) continue to identify specific risk loci, the polygenic nature of AD suggest the possibility that informative genetic signals may fall beneath the genome-wide significance thresholds. One approach to capturing these weak associations is to construct polygenic risk scores (PRSs) by taking the sum of all putative risk variants, defined broadly, weighted by their effect size from independent GWAS, assigning each individual a score, and then testing the association of this score with diagnosis and related phenotypes (8). For genetically complex diseases such as AD, PRSs have been shown to strengthen AD diagnostic classification beyond the use of *APOE* genotypes (9) and have further been shown to be associated with brain structure, Aβ and tau pathology, and cognitive decline (10–13). AD PRSs have also been shown to be associated with increased risk for mild cognitive impairment among individuals in their 50s (14), and have even been shown to be associated with brain structure in young adults (15,16), demonstrating their utility across multiple age ranges.

A benefit of PRSs is that they provide a global summary measure that aggregates the large and small effect sizes of different variants across the genome. However, aggregating the effects of individual loci may obscure distinct sources of risk. One approach to overcome this limitation has been to calculate PRS with the *APOE* region removed to demonstrate effects of the *APOE* gene and *APOE*-independent variants (17–19). In this light, AD risk genes identified through GWAS have been associated with a number of pathways, such as immune function, cholesterol transport, mitochondrial function, protein-lipid complex, and endocytosis (4, 6, 7, 20, 21), and the *APOE* gene itself impacts a variety of processes (22). Two individuals may therefore have similar scores on a global PRS with very different risk associated with the underlying pathways that are perturbed as a result. It is well documented that AD demonstrates substantial heterogeneity with respect to its clinical presentation (23–25), but also in the distribution of associated pathology and atrophy. For example, potential subtypes have been identified using AD-related biomarkers, including those associated with amyloid (26), tau (27–30), and neurodegeneration (31–35).

Breaking down global AD PRS into pathway-specific PRSs may be one approach to better understand the etiology of AD and its heterogeneity. Several studies have examined pathway-specific PRS in the context of AD but have typically focused on associations with diagnostic status or considered only genome-wide significant variants (19, 36–39). However, previous studies have highlighted the importance of including larger numbers of variants, including those below the level of genome-wide significance in relevant analyses (9, 14). In addition, there is a growing interest in characterizing individuals based on AD-related pathology involving amyloid, tau and neurodegeneration (i.e., the A/T/N classification system) (40) that are also likely to have a polygenic basis. As a result, we have examined associations between pathway-specific PRS with dementia status and A/T/N biomarkers to better characterize genetic influences on each.

## Methods

### Participants

Data used in the preparation of this article were obtained from the Alzheimer’s Disease Neuroimaging Initiative (ADNI) database (adni.loni.usc.edu). The ADNI was launched in 2003 as a public-private partnership, led by Principal Investigator Michael W. Weiner, MD. The primary goal of ADNI has been to test whether serial magnetic resonance imaging (MRI), positron emission tomography (PET), other biological markers, and clinical and neuropsychological assessment can be combined to measure the progression of MCI and early AD.

Our analyses included data on 1,411 participants from the ADNI-1 (n = 699), ADNI-GO/2 (n = 406), and ADNI-3 (n = 306) cohorts of European ancestry. The individuals in these cohorts had genome-wide genotype data that underwent rigorous quality control filters. Our analyses focused on cognitively unimpaired (CU) participants, participants with mild cognitive impairment (MCI), and individuals with AD dementia according to baseline ADNI diagnosis in the cohorts. Procedures were approved by the Institutional Review Board of participating institutions and informed consent was obtained from all participants.

### Genotype data processing and imputation

Individuals in the ADNI cohorts were genotyped using the following chips: Illumina Human610-Quad BeadChip (ADNI-1), Illumina HumanOmniExpress BeadChip (ADNI-GO/2), and Illumina Infinium Global Screening Array v2 (ADNI-3). Genetic data for each chip were processed and underwent quality control separately using PLINK2 (41) prior to genotype imputation. Single nucleotide polymorphisms (SNPs) were excluded if minor allele frequency < 0.05, sample missingness > 5%, or showed significant deviation from Hardy-Weinberg equilibrium (p < 1x10e − 06). Samples with genotype missingness > 10% were excluded. Participants were restricted to those with primarily European ancestry (> 80%) as determined by SNPweights (42). Principal components were calculated from linkage disequilibrium (LD)-pruned variants in combination with 1000 Genomes data (43) for use as covariates in later analyses. Imputation was performed on the Michigan Imputation Server (https://imputationserver.sph.umich.edu/) (44) using the 1000 Genomes phase 3 EUR reference panel. Imputed data from all phases was then merged.

### Identification of Alzheimer’s-related pathway clusters

A gene-set analysis of the IGAP2 Alzheimer’s disease GWAS summary statistics (4) was conducted with MAGMA v1.09a (45). SNPs were mapped to protein coding genes with a 35-kb upstream/10-kb downstream window. Gene sets from Gene Ontology (GO) (46, 47), Reactome (48) and KEGG (49) databases were downloaded from the Bader Lab website (http://baderlab.org/GeneSets). Analysis was restricted to gene sets containing 10 to 1,000 genes.

The resulting gene sets contain a high degree of redundancy, so we generated “pathway clusters” comprising gene sets with a high proportion of overlapping genes. First, the Cytoscape app EnrichmentMap v3.3 (50) was used to generate networks with gene sets as nodes and proportion of overlapping genes as edges. A gene set threshold of FDR q < 0.25 and overlap threshold of 0.5 were used as node and edge parameters, respectively. A permissive threshold was chosen to consider a broader set of potential risk pathways. Next, the AutoAnnotate app (http://baderlab.org/Software/AutoAnnotate) was used to cluster nodes with the MCL Cluster algorithm. Variants in genes belonging to a pathway cluster were used to calculate pathway-specific PRSs.

### Pathway-specific polygenic risk scores

Pathway-specific PRSs were calculated based on summary statistic effect sizes from the (4) Alzheimer’s disease GWAS using the PRSet function in PRSice-2 v2.3.5 (51). Each pathway PRS was calculated from SNPs mapped to genes contained in each pathway cluster with a 35-kb upstream/10-kb downstream window. A global PRS considering all SNPs contained in the summary statistics was also calculated. Prior to scoring, SNPs with MAF < 0.01 and imputation quality R^2^ < 0.5 were excluded from the analysis. LD clumping (r^2^ threshold of 0.2 in a 500 kb window) based on LD patterns in the 1000 Genomes EUR cohort was used to restrict scoring to independent loci. To determine the effect of PRSs independent of *APOE*, the region surrounding the *APOE* genes was removed (chr19:45,116,911–46,318,605 according to GRCh37). All remaining SNPS (i.e., p < 1) were used in scoring.

### Measures of amyloid, tau, and neurodegeneration

We explored associations between pathway-specific PRS and CSF and PET measures of amyloid and tau. We considered the results of CSF and PET measures as both are indicative of the presence of pathology, so we combined classifications from each to maximize the sample size for our analyses. Individuals were classified as Aβ and tau-positive based on having abnormal levels from at least one CSF or PET measure at a given data collection timepoint (e.g., a classification of Aβ + could be based on abnormal levels of amyloid from either a CSF or PET assessment, or both).

Aβ and p-tau CSF samples were collected on cohort participants and processed as previously described (52). CSF Aβ_42_ and p-tau were measured with the fully automated Elecsys immunoassay (Roche Diagnostics) by the ADNI biomarker core (University of Pennsylvania). Established cutoffs designed to maximize sensitivity in the ADNI study population were used to classify biomarker positivity [Aβ+: Aβ_42_ < 977 pg/mL; p-tau+: p-tau > 21.8 pg/mL] (http://adni.loni.usc.edu/methods) (53).

Aβ and tau PET data were processed according to previously published methods (http://adni.loni.usc.edu/methods) (54, 55). For Aβ, mean standardized uptake value ratios (SUVR) were created from a set of regions including frontal, temporal, parietal and cingulate cortices using whole cerebellum (florbetapir) or cerebellar gray matter (PIB) as a reference region. We used established cutoffs to determine Aβ-positivity for PIB-PET (SUVR > 1.44), florbetapir-PET (SUVR > 1.11), and florbetaben-PET (SUVR > 1.08) (54, 56). Partial-volume corrected flortaucipir (AV-1451) tau PET SUVRs were created from a meta-temporal region of interest that included amygdala, entorhinal cortex, fusiform gyrus, inferior temporal gyrus, and middle temporal gyrus regions using inferior cerebellar gray matter as a reference region. A cut-off of SUVR > 1.78 was used to define tau-PET positivity (57).

Neurodegeneration was indexed by the ratio of hippocampal volume to intracranial volume. As there is no established cut-off for abnormal hippocampal volume, this was used as a continuous measure in all analyses.

## Statistical analysis

All analyses were conducted with R v 4.2.1 (58). Differences in demographic variables were tested with t-tests for continuous variables and chi-squared tests for categorical variables. Associations with baseline diagnostic status restricted to cognitively unimpaired (CU) participants and those with dementia at their baseline visit and were tested using logistic regressions with diagnosis (CU vs dementia) as outcome. Associations with A/T/N biomarkers included participants of all diagnostic categories and used the first timepoint at which biomarker measures from all three categories were available. For amyloid and tau, our logistic regression models took biomarker abnormality status (positive vs negative) as the dependent or outcome measure of interest. For hippocampal volume, linear regression used the ratio of hippocampal volume to ICV as the outcome. Magnet field strength was also included as a covariate in analyses of hippocampal volume. Separate models were run with each PRS as predictor. The effect of *APOE* was assessed by including number of *APOE*-ε4 alleles (0, 1, or 2) as a separate variable. Models additionally included age, gender, and the first three principal components of the ADNI cohort genetic relationship matrix to control for any cryptic population stratification (59). Several post-hoc analyses were run to provide additional context to the PRS effect. Participants were stratified by *APOE*-ε4 into carriers and non-carriers, and PRS effects were tested separately in each group. Differences between these groups were directly tested with an interaction between *APOE*-ε4 carrier status and pathway PRS. To determine whether pathway-specific PRS in aggregate would increase predictive power, we fit models for each outcome (i.e., clinical status and biomarker abnormality) that included all pathway-specific PRS in the same model. The fit of these models were compared to models that included only the global PRS using Vuong’s likelihood ratio test (60). We corrected for multiple comparisons using Benjamini-Hochberg false discovery rate (FDR)-adjustment (61).

## Results

### Pathway analysis

The results of MAGMA analysis of the Alzheimer’s GWAS summary statistics suggested that several pathways were significantly enriched among associated variants – all surviving FDR correction for multiple comparisons (**Supplementary Table S1**). These included negative regulation of amyloid precursor protein catabolic process (GO:1902992), regulation of aspartic-type peptidase activity (GO:1905245), negative regulation of cellular component organization (GO:0051129), negative regulation of amyloid-beta formation (GO:1902430), and regulation of humoral immune response mediated by circulating immunoglobulin (GO:0002923). A number of other genesets were nominally significant (p < 0.05, uncorrected) and those with FDR q < 0.25 were included in clustering (**Supplementary Table S1**). Clustering yielded 13 pathway clusters: protein localization (including regulation of amyloid-beta and tau protein kinase activity), cholesterol transport, amyloid protein processing, immune signaling, inflammatory response (including microglial activation), endocytosis and fibril regulation, humoral immune response (including regulation of complement activation), receptor metabolic process, responses to misfolded protein, phototransduction, regulation of cell junction assembly, regulation of protein tyrosine, and mitophagy. Variants in the enriched pathways were used to construct the pathway-specific PRS. **Supplementary Table S2** lists the gene sets included in each of the pathway clusters.

### Associations with diagnostic status

**Figure 1:**
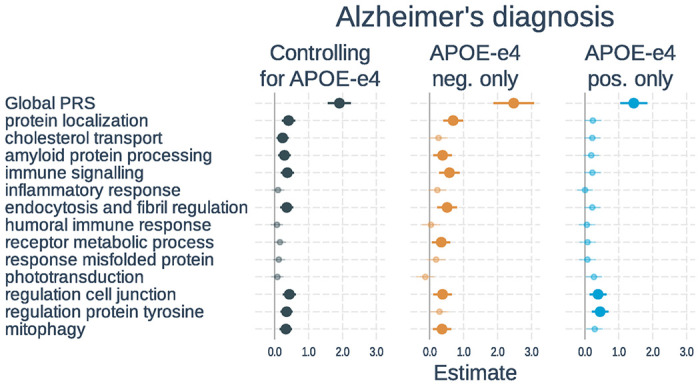
Associations of polygenic risk scores with diagnostic status. Logistic regressions were used with diagnostic status (cognitively unimpaired vs dementia) as the outcome. Separate models used each PRS as predictor. Models either 1) included number of *APOE*-ε4 alleles as a separate variable, 2) tested only *APOE*-ε4 non-carriers, or 3) tested only *APOE*-ε4 carriers. All models adjusted for age, gender, and the first 3 genetic principal components. Plots show standardized regression coefficients (log-odds) and standard errors. Associations that survived FDR correction are bolded.

Sample characteristics of participants included in the analysis of diagnostic status are listed in Table 1, and full results are shown in Fig. 1 and **Supplementary Table S3**. In models including PRS and *APOE*-ε4 status, a higher global PRS (β = 1.91, t-value = 11.01, p < 0.001) and number of *APOE*-ε4 alleles (β = 0.94, t-value = 8.60, p < 0.001) were significantly associated with an Alzheimer’s dementia diagnosis. Eight of the 13 pathway PRSs were significantly associated with diagnostic status after correction for multiple comparisons. These included: protein localization, cholesterol transport, amyloid protein processing, immune signaling, endocytosis and fibril regulation, regulation cell junction, regulation protein tyrosine, and mitophagy. When examining *APOE*-ε4 non-carriers only, results were similar except that cholesterol transport and regulation protein tyrosine were no longer significant whereas the receptor metabolic process PRS went from non-significant to significant. In *APOE*-ε4 carriers, only the global, regulation cell junction, and regulation protein tyrosine PRSs were significant. The effects of the global PRS (β=−0.54, t-value=−2.10, p = 0.035) and protein localization PRS (β=−0.45, t-value=−2.41, p = 0.016) were significantly weaker in *APOE*-ε4 carriers than non-carriers, whereas the phototransduction PRS was stronger in *APOE*-ε4 carriers (β = 0.39, t-value = 2.17, p = 0.030). However, these did not survive correction for multiple comparisons.

### Associations with amyloid positivity

**Figure 2:**
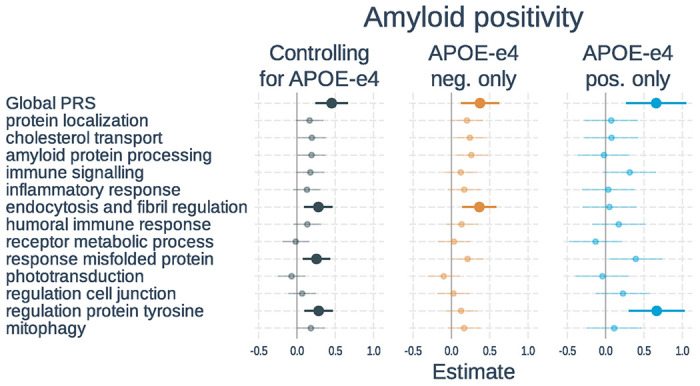
Associations of polygenic risk scores with amyloid positivity. Logistic regressions were used with amyloid status (positive vs negative) as the outcome. Separate models used each PRS as predictor. Models either 1) included number of *APOE*-ε4 alleles as a separate variable, 2) tested only *APOE*-ε4 non-carriers, or 3) tested only *APOE*-ε4 carriers. All models adjusted for age, gender, and the first 3 genetic principal components. Plots show standardized regression coefficients (log-odds) and standard errors. Associations that survived FDR correction are bolded.

Sample characteristics of participants included in the analysis of biomarkers are listed in Table 2, and full results of the associations with amyloid positivity are shown in Fig. 2 and **Supplementary Table S4**. In models including PRS and *APOE*-ε4 status, a higher global PRS (β = 0.45, t-value = 4.21, p < 0.001) and number of *APOE*-ε4 alleles (β = 1.14, t-value = 9.18, p < 0.001) were significantly associated with amyloid positivity. Three of the 13 pathway PRSs were significantly associated with amyloid status after correction for multiple comparisons. These included: endocytosis and fibril regulation, response misfolded protein, and regulation protein tyrosine. When examining *APOE*-ε4 non-carriers only, the global PRS as well as the endocytosis and fibril regulation PRS were significant. In *APOE*-ε4 carriers, only the global and regulation protein tyrosine PRSs were significant. The effect of the regulation protein tyrosine PRS was significantly stronger in *APOE*-ε4 carriers than non-carriers (β = 0.52, t-value = 2.55, p = 0.010), but this did not survive correction for multiple comparisons.

### Associations with tau positivity

**Figure 3:**
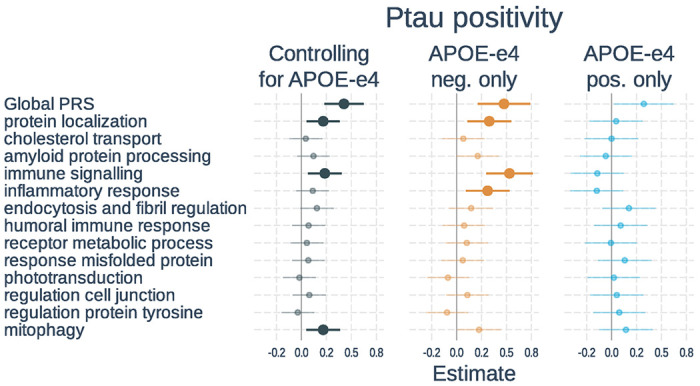
Associations of polygenic risk scores with ptau positivity. Logistic regressions were used with ptau status (positive vs negative) as the outcome. Separate models used each PRS as predictor. Models either 1) included number of *APOE*-ε4 alleles as a separate variable, 2) tested only *APOE*-ε4 non-carriers, or 3) tested only *APOE*-ε4 carriers. All models adjusted for age, gender, and the first 3 genetic principal components. Plots show standardized regression coefficients (log-odds) and standard errors. Associations that survived FDR correction are bolded.

Full results of the associations with tau positivity are shown in Fig. 3 and **Supplementary Table S5**. In models including PRS and *APOE*-ε4 status, a higher global PRS (β = 0.43, t-value = 4.25, p < 0.001) and number of *APOE*-ε4 alleles (β = 0.62, t-value = 6.63, p < 0.001) were significantly associated with tau positivity. Three of the 13 pathway PRSs were significantly associated with tau status after correction for multiple comparisons. These included: protein localization, immune signaling, and mitophagy. When examining *APOE*-ε4 non-carriers only, results were similar except that the mitophagy PRS was no longer significant, whereas the inflammatory response PRS became significant. In *APOE*-ε4 carriers, neither the global PRS nor any of the pathway PRSs were significant. The effect of the immune signaling PRS was significantly weaker in *APOE*-ε4 carriers than non-carriers (β=−0.66, t-value=−3.72, p < 0.001), but this did not survive correction for multiple comparisons.

### Associations with hippocampal volume

**Figure 4:**
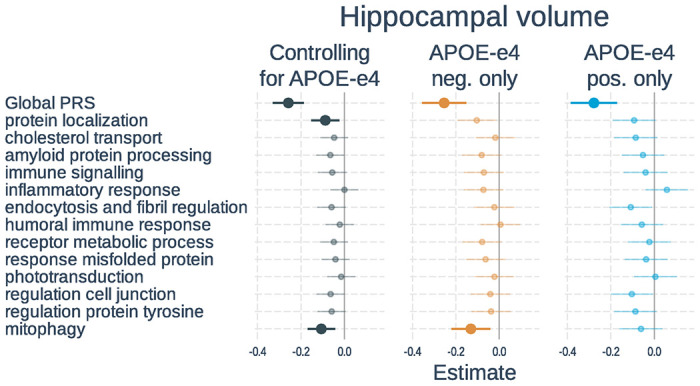
Associations of polygenic risk scores with hippocampal volume. Linear regressions were used with hippocampal volume normalized by intracranial volume as the outcome. Separate models used each PRS as predictor. Models either 1) included number of *APOE*-ε4 alleles as a separate variable, 2) tested only *APOE*-ε4 non-carriers, or 3) tested only *APOE*-ε4 carriers. All models adjusted for age, gender, scanner field strength and the first 3 genetic principal components. Plots show standardized regression coefficients and standard errors. Associations that survived FDR correction are bolded.

Full results of the associations with hippocampal volume are shown in Fig. 4 and **Supplementary Table S6**. In models including PRS and *APOE*-ε4 status, a higher global PRS (β=−0.26, t-value=−6.88, p < 0.001) and number of *APOE*-ε4 alleles (β=−0.24, t-value=−6.98, p < 0.001) were significantly associated with smaller hippocampal volume. Two of the 13 pathway PRSs were significantly associated with tau status after correction for multiple comparisons. These included: protein localization and mitophagy. When examining *APOE*-ε4 non-carriers only, the global PRS and mitophagy PRS were significant. In *APOE*-ε4 carriers, only the global PRS was significant. The PRS effects were not significantly different between *APOE*-ε4 carrier and non-carriers.

## Discussion

The current results support and extend previous work disentangling the biological pathways contributing to Alzheimer’s disease risk and pathogenesis. Consistent with previous findings, we found that a global AD PRS was significantly associated with diagnostic status, amyloid and tau positivity, and hippocampal volume (9–13, 15, 17). The global AD PRS captures the combined effects of multiple separable influences on disease risk and therefore is not useful for teasing apart genetically-mediated etiological differences among individuals. Several studies have examined the relationship of AD diagnosis or AD-related biomarkers with pathway-specific PRS calculated from GWAS-significant SNPs (36, 37, 39). Here, we generated pathway-specific PRSs from clusters of gene sets and SNPs with association strength p-values falling below the threshold of GWAS significance. Breaking down the global effects of polygenic factors into more refined genetic pathway-associated subset of polygenes has been shown to provide useful information above-and-beyond variants with more pronounced effects arising from GWAS for certain conditions, including AD and MCI (8, 9, 14).

As expected, most of the pathway PRSs associated with AD diagnostic status in the current study correspond to pathways that have consistently been uncovered in previous GWAS. These pathways include amyloid precursor processing, immune and microglial response, endocytosis, cholesterol transport, lipid-protein complex and amyloid clearance (4, 6, 7, 21). Several pathways of focus in our study, however, had less support from other GWAS, but have nonetheless been linked to AD in yet other studies. For example, the regulation cell junction and regulation protein tyrosine pathway-specific PRS we considered may capture effects of genes involved in synaptic functioning and cell signaling, consistent with studies suggesting this pathway is involved in AD-related cognitive decline (62, 63). In addition, mitochondrial function has been proposed as playing a key role in the development of AD (64, 65), and mitophagy in particular may have widespread impacts on age-related disease, including Alzheimer’s disease (66). Our results are also consistent with studies that have examined the association of pathway-specific PRS with AD diagnosis, including Aβ clearance, cholesterol transport, immune response, and endocytosis (19, 36, 37, 39)

Amyloid positivity was significantly associated with pathway-specific PRS for endocytosis and fibril regulation, response to misfolded proteins, and regulation of protein tyrosine. The pathways associated with these PRSs are involved in the production, trafficking and clearance of Aβ peptides, as well as their aggregation into fibrils, which has biological plausibility. The endocytic pathway plays a key role in the amyloidogenic processing of APP as it is internalized to the intracellular space followed by cleavage into Aβ in the early endosome (67, 68). Tyrosine kinases may be involved in both the trafficking of APP and upregulating BACE activity (69, 70). In addition, genes encompassed by the response misfolded protein PRS that we find associated with AD pathology, include molecular chaperones (e.g., CLU) and the ubiquitin-proteasome system, which mediate degradation of abnormal and misfolded proteins (71–73). A previous study examining pathway-PRS found that PRS related to Aβ clearance and cholesterol metabolism were also related to CSF and PET measures of amyloid; however, these scores only included GWAS-significant variants and the effects were primarily driven by *APOE*(37).

We also found that tau positivity was significantly associated with pathway PRSs for protein localization, immune signaling, and mitophagy. The protein localization pathway includes tau protein kinase activity, which may relate to abnormal hyperphosphorylation of tau (74). Sun et al. (75) also found that a PRS reflecting the tau kinase activity was associated with CSF and PET measures of tau. Tau binds to microtubules to provide stabilization, but detaches when phosphorylated which can reduce axonal integrity and disrupt protein transport along the cytoskeleton (76). Additionally, this unbound phosphorylated tau can aggregate into neurofibrillary tangles (77). Regarding the PRS reflecting immune signaling, although inflammation can be secondary to abnormal tau, there is also evidence for an upstream role of glial activation and neuroinflammation in driving the accumulation and spread of tau (78, 79). This is consistent with a previous finding that a PRS constructed from only GWAS significant variants related to immune response was associated with CSF tau (37). As with inflammation, disruptions to mitophagy may be secondary to disease processes, but there is evidence for upstream roles of mitochondrial function, including mitophagy, in the development of AD pathology (65, 80). Hippocampal volume was also associated with pathway PRSs for protein localization and mitophagy. Shared pathways with tau positivity and may reflect the tighter linkage between neurodegeneration and tau compared to amyloid (81, 82).

Our results are generally consistent with those of a recent GWAS on CSF measures of amyloid and tau (83). The authors found little overlap in the loci associated with amyloid and tau aside from *APOE*. In contrast, there was overlap between loci associated with tau and ventricular volume, which can be used as an indicator of neurodegeneration. The authors also examined associations of AD-associated variants with CSF amyloid and tau, and a cluster analysis pursued by them identified patterns that were broadly similar to our own. For example, variants associated with CSF amyloid were associated with amyloid processing, endocytosis, and tyrosine kinase, whereas variants associated with CSF tau were linked to the immune system. Deriving PRSs based on GWAS of AD biomarkers represents a promising approach to index risk in specific pathways, but sample sizes for such studies are relatively small. PRSs based on pathway analysis of diagnosis-based GWAS are therefore a useful alternative that can leverage large case-control datasets to provide converging information.

Although we focused on *APOE*-independent sources of AD risk, it is important to note that the number of *APOE*-ε4 alleles had a comparable or even stronger (in the case of amyloid) effect on the outcomes as the polygenic component indexed with a PRS. *APOE* belonged to gene sets that were part of several cluster, including endocytosis and fibril regulation, protein localization, cholesterol transport, and amyloid protein processing. The variants falling within the *APOE* region were excluded from our PRSs, but this does suggest *APOE* can exert an impact through multiple routes. Stratifying based on *APOE*-ε4 carrier status suggested stronger effects of pathway PRSs on tau positivity in non-carriers. It may be that smaller polygenic effects are obscured in the presence of a larger *APOE*-related signal. Alternatively, *APOE*-ε4 may be sufficient to increase risk for tau pathology whereas those lacking this risk allele require additional sources of risk to develop abnormal levels of tau.

There are several additional items worth mentioning to put our analyses into context. First, we find evidence that the effect size of the global PRS is much larger than any pathway-specific PRS effect sizes in our analyses. While pathway-specific PRSs may be beneficial for understanding disease etiology, they do not appear to add predictive power when considered in the aggregate over-and-above the global PRS. Second, we mapped SNPs to genes using the standard position-based approach available in MAGMA. However, many GWAS SNPs are located in non-coding regions and may be associated with disease risk through their gene regulatory effects (84). Thus, approaches that make use of information such as chromatin interactions (85) or expression quantitative trait loci (86) to determine which gene a variant present in a non-coding region affects may prove useful in developing pathway-specific PRS. Third, genes/SNPs may be part of multiple pathway clusters, so the pathway PRSs are not entirely independent with each other. Fourth, we added interactions with diagnosis to all biomarker models to determine whether PRS associations differed by group. However, no interaction terms were significant after correcting for multiple comparisons and there did not appear to be a consistent pattern among the few interactions that reached nominal significant. Finally, this analysis required choices of parameters at various steps, such as the p-value thresholds used to filter variants and gene sets, method used to construct PRS, and even which gene sets were used. We believe our choices represent a reasonable attempt to capture the broad sources of polygenic influence on AD risk while minimizing unrelated signal. However, alternative approaches may be equally valid and should be determined by the context of a given analysis.

## Conclusions

Ultimately, we find evidence that some pathway-specific PRSs are associated with AD diagnostic status and A/T/N biomarkers. Our findings indicate that genetic risk for AD may exist along multiple dimensions, and the distribution of risk across pathways may influence phenotypic manifestations of the disease. Although a global PRS appears to provide superior predictive power overall, pathway-specific PRS analysis may help clarify aspects of the heterogeneity of AD pathogenesis.

## Figures and Tables

**Table 1:** Characteristics of sample used to test associations with diagnostic status.

	Cognitively Unimpaired	Dementia
n	536	214
Gender (Male), n (%)	258 (48.1)	122 (57.0)
Age, mean (SD)	73.49 (5.98)	75.46 (8.11)
Years of education, mean (SD)	16.56 (2.50)	15.11 (2.96)
*APOE*-ε4 carrier, n (%)	161 (30.0)	143 (66.8)

**Table 2:** Characteristics of sample used to test associations with biomarker measures.

	Biomarker sample
n	674
Gender, n Male (%)	387 (57.4)
Age, mean (SD)	73.77 (7.36)
Years of education, mean (SD)	15.97 (2.76)
*APOE*-ε4 carrier, n (%)	300 (44.5)
Diagnosis, n (5)	
CU	200 (29.7)
MCI	373 (55.3)
Dementia	101 (15.0)
Amyloid positive, n (%)	430 (63.8)
Ptau positive, n (%)	382 (56.7)
Hippocampal volume ratio, mean (SD)	0.44 (0.08)

CU = cognitively unimpaired, MCI = mild cognitive impairment. Hippocampal volume ratio was calculated as (Hippocampal volume / Intracranial volume)*100.

## Data Availability

Data used in this article can be downloaded from the Alzheimer’s Disease Neuroimaging Initiative (ADNI) database at adni.loni.usc.edu.

## Abbreviations

AD: Alzheimer’s disease
ADNI: Alzheimer’s Disease Neuroimaging Initiative
A/T/N: amyloid/tau/neurodegeneration
CSF: cerebro-spinal fluid
CU: cognitively unimpaired
FDR: false discovery rate
GWAS: genome-wide association studies
LD: Linkage disequilibrium
MCI: mild cognitive impairment
MAF: minor allele frequency
MRI: magnetic resonance imaging
PET: positron emission tomography
PRS: polygenic risk score
SNP: Single nucleotide polymorphisms
SUVR: Standardized uptake value ratio
